# Fragments of Trade and Consumption: Plant Macroremains from the Boa Vista Ships in Lisbon

**DOI:** 10.1007/s11457-026-09498-2

**Published:** 2026-02-09

**Authors:** Mariana Costa Rodrigues, José António Bettencourt, João Pedro Tereso, Dulce Freire, Inês Mendes da Silva

**Affiliations:** 1 CHAM – Centre for the Humanities, NOVA University of Lisbon, Lisbon, Portugal; 2 IEM – Institute for Medieval Studies, NOVA University of Lisbon, Lisbon, Portugal; 3 CEIS20 – Centre for Interdisciplinary studies, Faculty of Economics of University of Coimbra, Coimbra, Portugal; 4 ERA Arqueologia S A – Centro de História Universidade de Lisboa, Lisbon, Portugal

**Keywords:** Archaeobotany, Atlantic trade, Early modern period, Cargo, On-board consumption, New foods

## Abstract

Archaeological interventions along Lisbon’s riverside have revealed critical insights into the city’s transformation from a maritime beach to an industrialized port zone. Excavations at the former Praia da Boa Vista revealed three ships (Boa Vista 1, 2, and 5), probably abandoned on the old Boa Vista beach between the last half of the seventeenth century and first half of the eighteenth century. These contexts, linked to the Atlantic trade and intense port activity, included botanical remains such as coconuts, cocoa, olives, and cherries, reflecting both crew consumption and commercial trade. Manual sampling favored large botanical materials, creating a preservation bias and limiting the interpretation of cargo diversity. Limitations included loss of stratigraphic data and label degradation. Nevertheless, the findings illuminate the integration of exotic and local products in Lisbon’s maritime economy. This study underscores the need for improved sampling methods in waterlogged environments and highlights how fragmentary remains can offer valuable insights into daily life, trade routes, and the environmental and commercial evolution of early modern Lisbon.

## Introduction

Lisbon played a central role in the introduction and circulation of plant-based products, serving as a crucial hub for their distribution throughout Europe and supporting their global cultivation. While this subject has been widely discussed in written sources (Flandrin and Montanari 1999; Crosby 2003; Freire 2020; Barros 2013; Gomes 2017; Ferrão 2005), archaeobotanical studies, especially concerning the Early Modern period, remain scarce. Lisbon was at the heart of food globalization, receiving, and distributing numerous economically significant plants. However, archaeological evidence of such materials has yet to be thoroughly investigated. Studies in other parts of Europe have demonstrated the potential of archaeobotany to enrich historical records, underscoring the need for further research in Lisbon (Larsson and Foley 2023; Martín-Seijo et al. 2021; Clapham 2003).

An ongoing research project aims to fill this significant gap in Portuguese archaeobotanical research by focusing on ship-associated materials from the Early Modern period. By integrating archaeological, archaeobotanical, and historical data, this research seeks to provide a more comprehensive understanding of the introduction and use of these botanical materials in Lisbon and beyond. The project aims to offer insights into the maritime trade and cultural routes that linked Lisbon with Africa, Asia, and the Americas, by tracing the movement and local presence of plant materials associated with vessels and other port contexts. The research focuses on the timeframe and on the paths by which specific fruits and nuts became part of Lisbon’s material and dietary culture, contributing to the broader understanding of biological globalization. This study is a step towards uncovering Lisbon’s role as a key node in the transcontinental exchange of food-related goods and materials during the early phases of globalization. It also aims to facilitate comparative research with other global port cities, helping to better understand the scale, impact, and rhythms of material and food globalization in the Early Modern period. Finally, it seeks to assess the socio-economic and cultural implications of these materials.

This article presents research showing that these materials provide clues about the city’s role within global food and trade networks. The paper is structured into interrelated sections that together offer a comprehensive view of the plant macroremains recovered from three Early Modern ships from Lisbon’s Boa Vista area. It opens with a summary of the main findings, followed by an introduction on Lisbon within global trade networks and highlighting the limited scope of archaeobotanical research in Portugal. The archaeological context and ship descriptions provide the foundation for the study, while the methodology section outlines sampling procedures and their limitations. The results are presented through comparative data from the three ships, and the discussion explores their historical and economic significance. The conclusion reflects on the broader implications of these findings for understanding Lisbon’s role in early global botanical circulation.

### The Archaeological Contexts

**Fig. 1:**
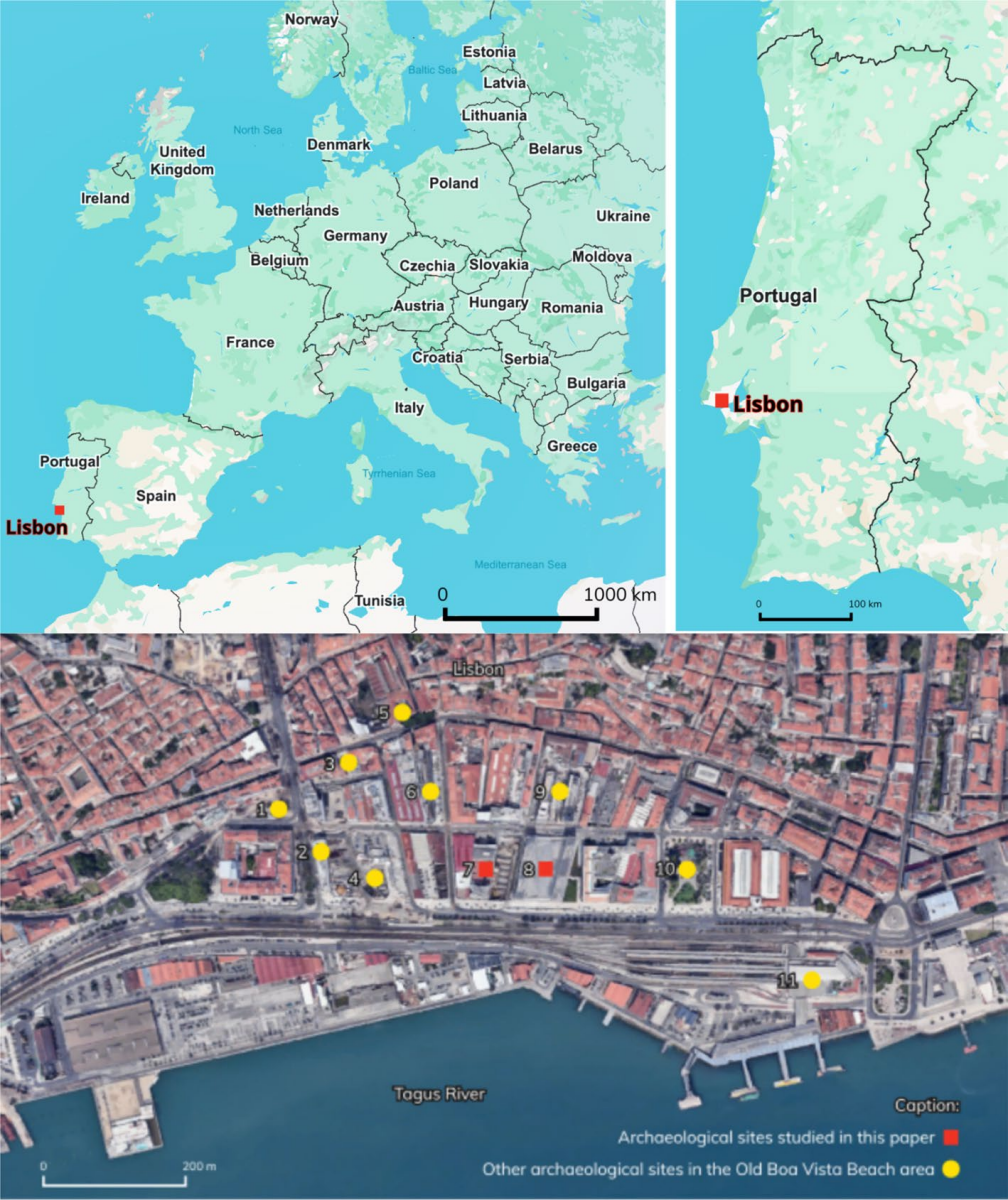
Location of Lisbon in Europe and Portugal and of the archaeological sites connected to the old Boa Vista Beach in Lisbon. Relevant archaeological sites for the study of the riverside evolution of the old Boa vista, modern period Beach area marked on the map (Google Earth): Vitorino Damásio Square (excavated in 2013)—wooden anchorage/mast and arsenal services/wooden structure using parts of boats for formwork – seventeenth/eighteenth century (Santos 2006). D. Carlos I Avenue (renovated in 2013)—masonry structure, possible dock/structures with wooden forms—seventeenth/eighteenth century (Gomes 2014). Cais do Tojo Street – Small planks and isolated finds of nautical pieces and other materials – seventeenth/nineteenth century / landfill and silting levels with isolated materials, including an anchor and a stump – seventeenth/eighteenth century (Simão et al. 2019). Lisbon’s metro line – Archaeological works still undergoing. Almada-Carvalhais Palace—Headquarters of the Casa da India, built in the sixteenth century—Recent interventions have identified contexts from recent prehistory to the twentieth century (ERA Arqueologia 2024). Boqueirão do Duro—possible stilt dock/wood storage areas/various nautical pieces—seventeenth/nineteenth century (Macedo et al. 2017). 24 de Julho Avenue/ Essentia/ Boa Vista 5 – Large vessel for oceanic navigation, varied artefactual component, seventeenth/eighteenth century (Mendes da Silva 2022). EDP 1—2 large vessels for Mediterranean/Atlantic navigation, varied artefactual component—seventeenth/eighteenth century (Bettencourt et al. 2014). EDP 2—Wooden structure, possible pier, and several small boats, varied artefactual components—seventeenth/nineteenth century (ERA Arqueologia 2022). D. Luís I Square- Roman anchorage, ramp or tidal grid with reused boat parts first century BC to fifth century AD; seventeenth/nineteenth century (Srrazola et al. 2017). Cais do Sodré—Large vessel—fifteenth/sixteenth century (Bettencourt et al. 2021a)

**Fig. 2:**
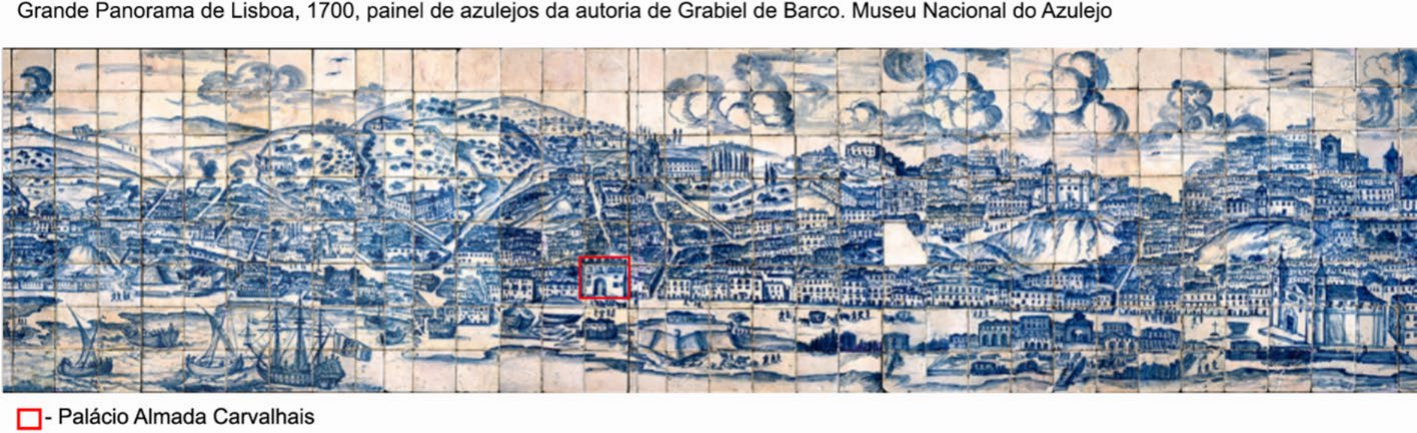
Tile panel depicting the riverfront before the 1755 earthquake. The Almada Carvalhais Palace can be seen on the riverbank, along with old Boa Vista beach, several vessels, and port structures

In recent decades, dozens of archaeological interventions have been carried out in the riverside area of Lisbon, in Santos, formerly occupied by the old Boa Vista beach (*Antiga Praia da Boa Vista*, in Portuguese) (Fig. 1). The sites found in that area have similar contexts, which document the evolution of the area from the Roman to modern periods. In general, the archaeological excavations uncovered a long stratigraphic sequence, that can be divided in into three main phases. The first level, which corresponds to port contexts, is located at depths of between 2 and 7 m (mean sea level), which preserves ceramics dating from Roman times to the eighteenth century, as well as various harbor structures, quays and ramps, nautical equipment’s, such as iron anchors, or abandoned ships. Above these levels, consisting of silty sediments, several landfills were built in the eighteenth and nineteenth century, sealing the port contexts and serving as the foundation for the construction of commercial and industrial buildings in the nineteenth and twentieth centuries (Bettencourt et al. 2021a). Santos and the old Boa Vista beach was until the fourteenth century an outskirt area. In the second half of that century there are references to the use of this area for maritime activities. It is, however, in the fifteenth and sixteenth centuries that the settlement of fishermen, sailors, and other groups with professions linked to maritime and commercial activities in this region intensified, forming the extramural neighborhoods of Boa Vista and Santos o Velho. In 1502, D. Manuel I, King of Portugal designated the old Boa Vista beach as a shipbuilding and maintenance area (Mendes da Silva 2025). Indeed, between 1502 and 1756, a series of provisions were enacted establishing that Boa Vista was to remain unobstructed, thereby ensuring unrestricted access for all ships and vessels. These measures prohibited any form of construction and mandated the demolition of existing structures identified along the shoreline. The *Provedor da Casa da Índia* (responsible for the administration, commerce, and customs of overseas territories controlled by Portugal) is installed here, in the *Almada Carvalhais* Palace (Macedo et al. 2017). The Palace was on the first line of the old Boa Vista beach as you can see in Fig. 2, clearly demonstrating the occupation of land over the river due to human action (landfills from the modern and contemporary era), visible if we observe the current location of the Palace (Fig. 1). In the seventeenth century, this area was occupied by the *Ribeira da Junta do Comércio* logistics (Board of Trade, a governing body that oversaw commercial and economic activities between Portugal and its overseas colonies, particularly Brazil, intended for ship repair and construction (Macedo et al. 2017). In the eighteenth century the port activity in the area intensified—a customs institution and the *Casa da Moeda* (Portugal’s official minting authority) were installed in São Paulo street, which had a private dock, the *Cais do Tojo* and the *Paço da Madeira* (1798) (Macedo et al. 2017). In the eighteenth century and beginning of the nineteenth century there were more than twenty shipyards and many warehouses (Macedo et al. 2017).

Among the archaeological deposits, which include open working areas on the beach, quays, ramps, embankments or landfills (Bettencourt et al. 2021a), are three ships excavated in the area formerly occupied by the old Boa Vista Beach – Boa Vista 1, Boa Vista 2, and Boa Vista 5, the subject of this paper.

### EDP 1 Archaeological Site, Boa Vista 1 and Boa Vista 2 Vessels

The remains identified in the EDP I project, during the construction of the Energias de Portugal headquarters, were excavated in 2012 and 2013 by ERA Arqueologia S A, in which CHAM—Centre for the Humanities (NOVA University of Lisbon) collaborated. The site revealed evidence of port activities, like pottery with chronologies varying between the Roman period and the eighteenth century, several Early Modern iron anchors, glassware, or organic elements, like leather and ropes. The deposits also preserved two ships with chronologies between the second half of the seventeenth century and the first half of the eighteenth century, named Boa Vista 1 and Boa Vista 2 (Bettencourt et al. 2014).

**Fig. 3:**
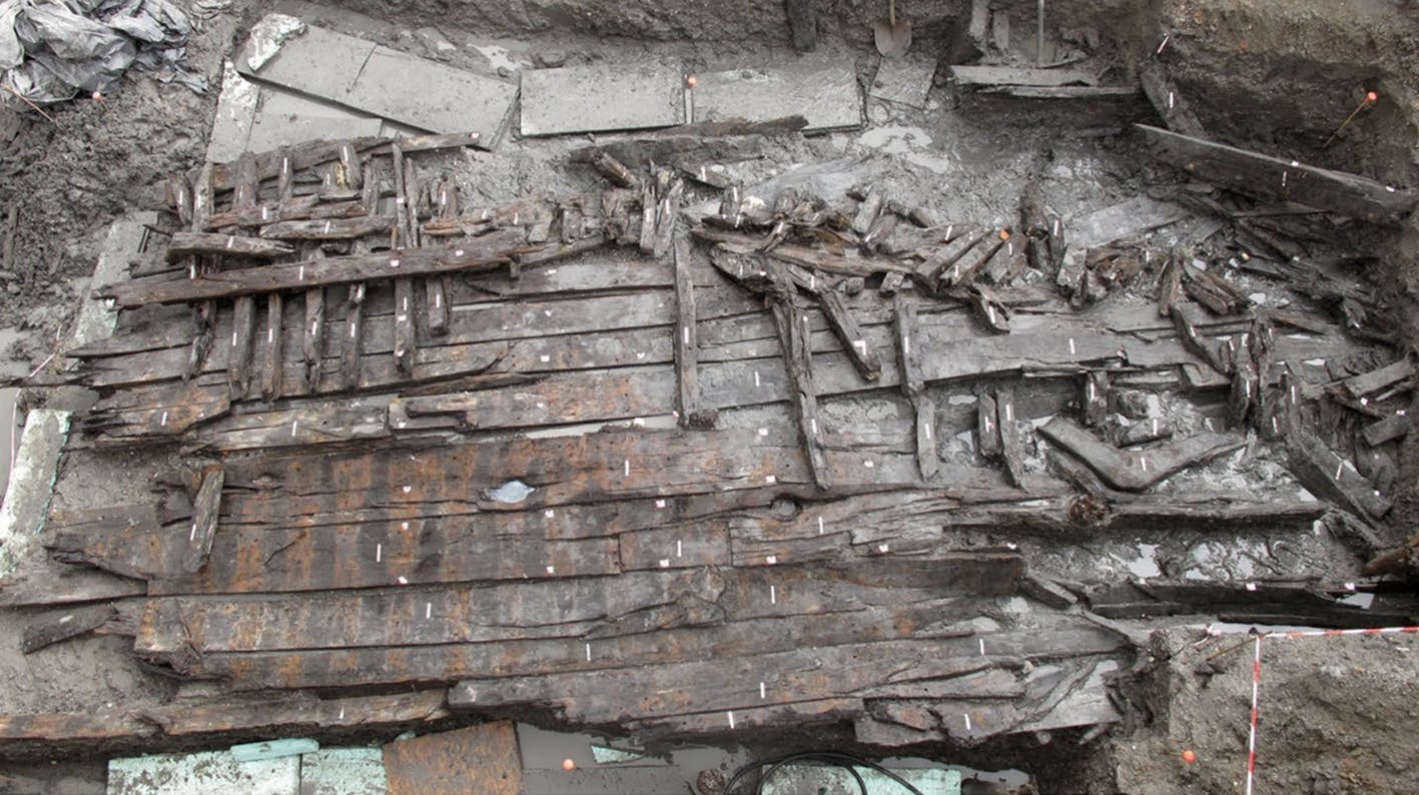
The Boa Vista 1 vessel during excavations (photo: CHAM)

**Fig. 4:**
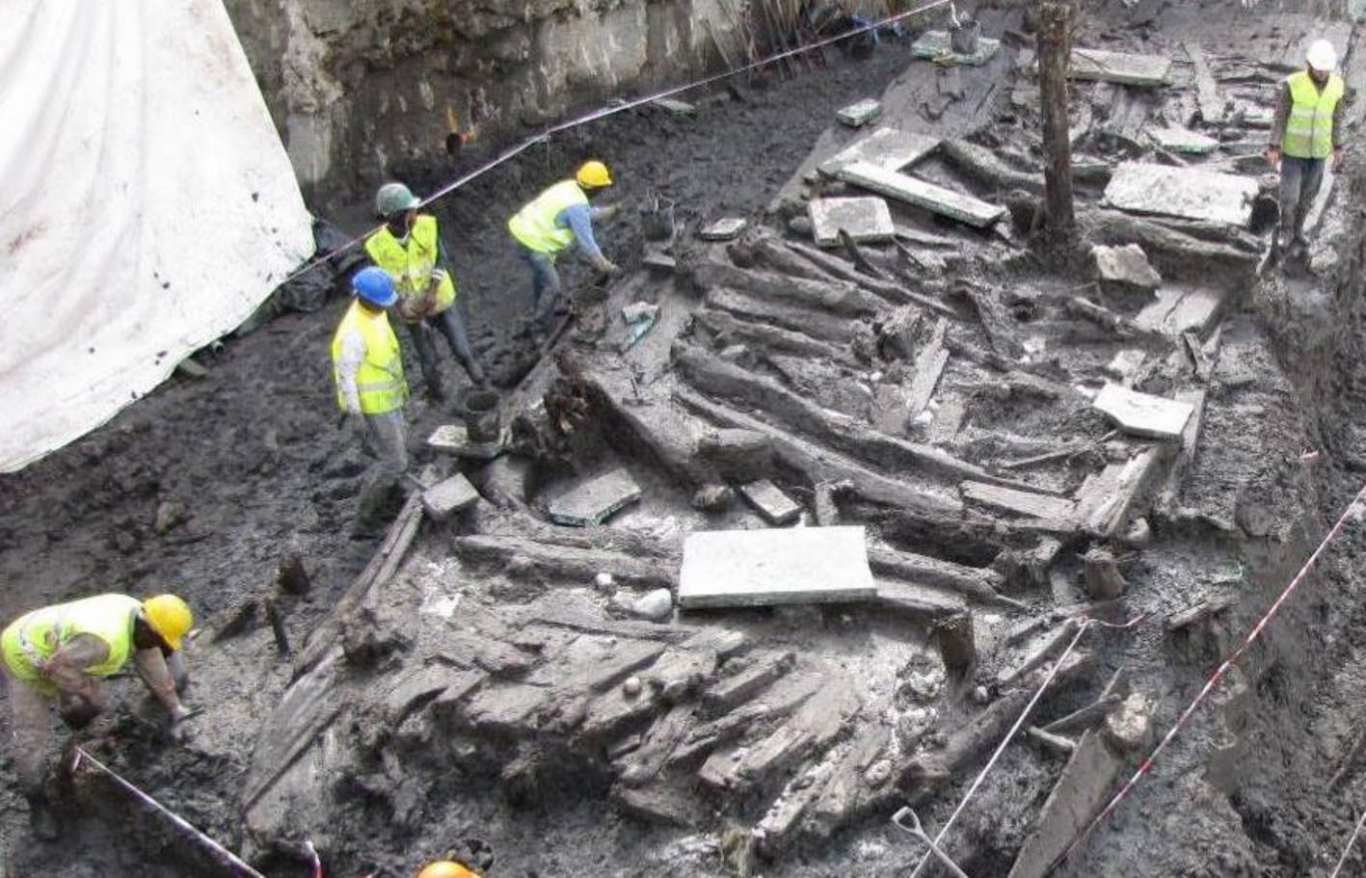
The Boa Vista 2 vessel during excavations (photo: ERA Arqueologia S A and CHAM archives)

As we have seen, the area where the ships were excavated corresponds to the old Praia da Boa Vista, submerged until the eighteenth century. The location and formation processes identified during the study of the two hulls suggest that they were partially dismantled and abandoned on the beach in the intertidal zone in the transition between the seventeenth to eighteenth centuries (Bettencourt et al. 2021a, 2014). Boa Vista 1 (Fig. 3), a small/medium-sized vessel, presents some characteristics common to the Mediterranean and others present in the Atlantic area, therefore presenting hybrid characteristics (Lopes 2022; Lopes Petrucci-Fonseca 2023; Bettencourt et al. 2021b). The larger Boa Vista 2 (Fig. 4) has no clear parallels in the available literature, although the context suggests that it was built in an area of Iberian influence, having operated on colonial routes, possibly in the Atlantic. Among its unique features is the use of a sacrificial lining placed over a layer of a whitish mixture, possibly the gala-gala mentioned in sixteenth-century Portuguese written sources (Bettencourt et al. in press; Bettencourt et al. 2021a).

**Fig. 5:**
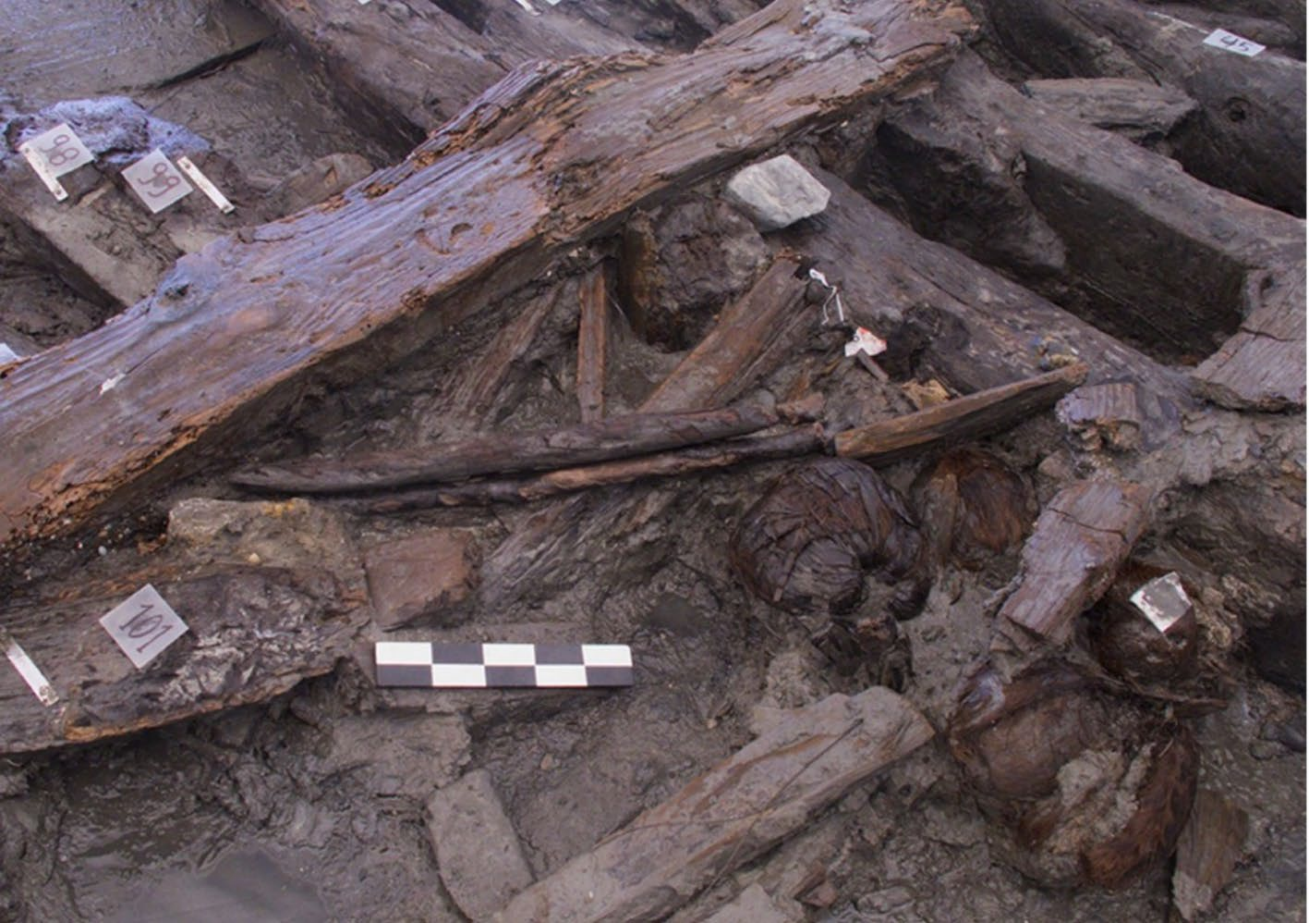
Coconuts stored in the hold of the Boa Vista 2 ship during excavation (photo: CHAM)

The two ships were sealed with silty sediments—stratigraphic units SU 1001 in Boa Vista 1 and SUs 2001/2002 in Boa Vista 2—containing seventeenth/eighteenth-century materials (Dutch or English kaolin pipes, English onion bottles, Rhenish stoneware and Portuguese faience), allowing the abandonment to be dated in this chronology. Most of these artifacts, however, cannot be associated with the ship but rather with the port activities that took place in this area (Bettencourt et al. 2021a). The deposits that covered the ships also preserved plant materials, namely coconuts, and others, most of which cannot be associated with the two ships either. The only exception is the collection of coconuts identified next to the hull of the Boa Vista 2, which would have been stored in the ship's hold (Fig. 5).

### 24 de Julho Avenue/ Essentia Arcaheological Site, Boa Vista 5 Vessel

**Fig. 6:**
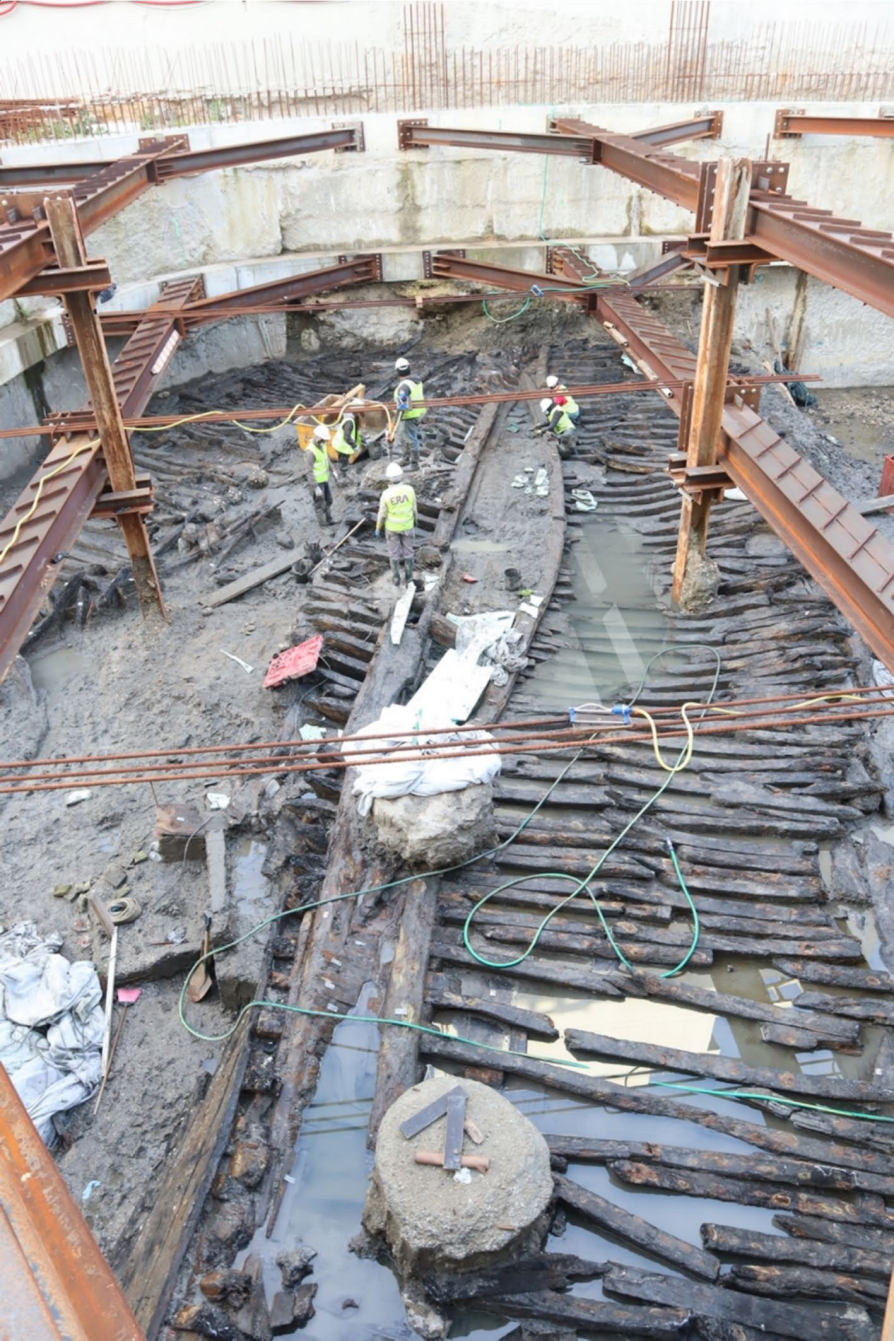
The Boa Vista 5 vessel during excavation (photo: CHAM)

Excavated in 2020–2021 in the block next to EDP I, the Boa Vista 5 (Fig. 6) is the best-preserved ship ever found in the Lisbon waterfront (Bettencourt et al. 2024). The ship was discovered on the northern edge of a former anchorage, abandoned on the beach, in the intertidal zone, sometime during the last quarter of the seventeenth century. The archaeological excavation uncovered a long stratigraphic sequence like that recorded during the excavations of the Boa Vista 1 and 2 vessels in the adjacent block (Bettencourt et al. 2021a, b). In this site, the port area, located at depths of between 2 and 7 m (mean sea level), preserved ceramics dating from the Roman period to the eighteenth century, as well as various nautical equipment, such as iron anchors or a small river boat, Boa Vista 4. Above these levels, several landfills were excavated, which sealed Boa Vista 5 and served as a base for the construction of commercial and industrial buildings in the nineteenth century Table 1.

**Table 1:** – Identification of macroremains from the Boa Vista 1 vessel. SU stands for stratigraphic unit

Boa Vista 1						
SU	Sector	*Cocos nucifera*		*Olea europea*		Total number of macroremains
		Unit	Fragment	Unit	Fragment	
1001	1149.12 AD BV1		1	2		**3**

When discovered, the ship was covered by a ballast mound, that dominated the central section of the hull. Artefacts found during the excavation and clearly related to the vessel are scarce, but include, for example, pulley pieces, round iron shot and coconuts and others plant materials found under and among the ballast. In addition to the botanical material and ship components, the collected objects included carnelian beads, stoneware bottles, kaolin pipes, Chinese crockery, among others (Mendes da Silva 2022). The plant remains studied at Boa Vista 5 were identified in four stratigraphic units (SU). SU 1488 corresponds to the ballast that covered the central section of the ship, later divided into quadrants during dismantling, which correspond to the alphanumeric code accompanying the data in Table 2. EU 1487 corresponds to the ship. SU 1494 corresponds to the silty to sandy levels that occupied the entire intervention area, covering the Boa Vista 5 ship and containing various materials from the eighteenth century. SU 1497 corresponds to the sedimentary levels under the ship, where carnelian beads, among other materials, were identified.

**Table 2:** – Identification of macroremains from the Boa Vista 2 vessel. SU stands for stratigraphic unit

Boa Vista 2														
SU	Sector	*Cocos nucifera*		*Prunus avium/ cerasus*		*Olea europea*		*Pinus pinea (pinion)*		*Cucumis melo*		*Vitis vinifera*		Total number of macroremains
		Unit	Fragment	Unit	Fragment	Unit	Fragment	Unit	Fragment	Unit	Fragment	Unit	Fragment	
2002	1162. AD BV2	4	8											**12**
2001/2002	1162. AD BV2	8	62	4						1		1		**76**
2001	1162. AD GMB2			4		1			1					**5**
	1162.12		1											**1**
2001	1162. AD BV2		5											**5**
2001/2002	1162.12 BV2		2											**2**
	Total	**12**	**78**	**8**		**1**			**1**	**1**		**1**		**102**

## Methodologies

### Sampling

This study includes only macroremains handpicked during excavation of Boa Vista 1, Boa Vista 2, and Boa Vista 5, which remain the predominant recovery method used at archaeological sites in Portugal, particularly for these chronologies. This is largely due to the absence of legislation mandating systematic soil sampling and the relatively recent integration of archaeobotany and environmental archaeology into university curricula; moreover, the operational constraints typical of preventive archaeology in densely built urban contexts—including restricted timeframes, reduced excavation areas, and pressures associated with construction schedules—further constrain the implementation of fine-grained sampling strategies essential for the systematic retrieval of plant macro-remains.

Due to logistical constraints, not all plant remains were collected in the field, with the exception of the macroremains found next to the hull. Manual recovery involves the direct collection of plant materials visible to the naked eye during excavation. These remains are collected similarly to other finds, such as ceramics, and are placed in separate containers marked with essential provenance data (site code, date, stratigraphic information, etc.). Containers are numbered and recorded in the field inventory. In the case of plant materials recovered from waterlogged contexts, the remains are preserved in water to prevent degradation.

This recovery method necessarily imposes a strong bias on the dataset. Hand-picking favors large, visually prominent remains, while smaller seeds and fragments often remain undetected, resulting in an overrepresentation of larger taxa and an underrepresentation—or complete absence—of smaller ones. This underscores the importance of implementing systematic sediment sampling in future excavations to obtain a more representative archaeobotanical assemblage. Archaeobotany remains a lesser-known field within the Portuguese archaeological community, but it is a growing subject with more studies and researchers dedicated to archaeobotany every year (Vaz et al. 2024). Ongoing efforts by archaeobotanical researchers in Portugal are focused on promoting standardized sediment-sampling practices and addressing logistical constraints faced by both public and private institutions. Sediment samples have been collected from comparable sites more recently and will be analyzed separately in future work.

### Laboratory Procedures

**Fig. 7:**
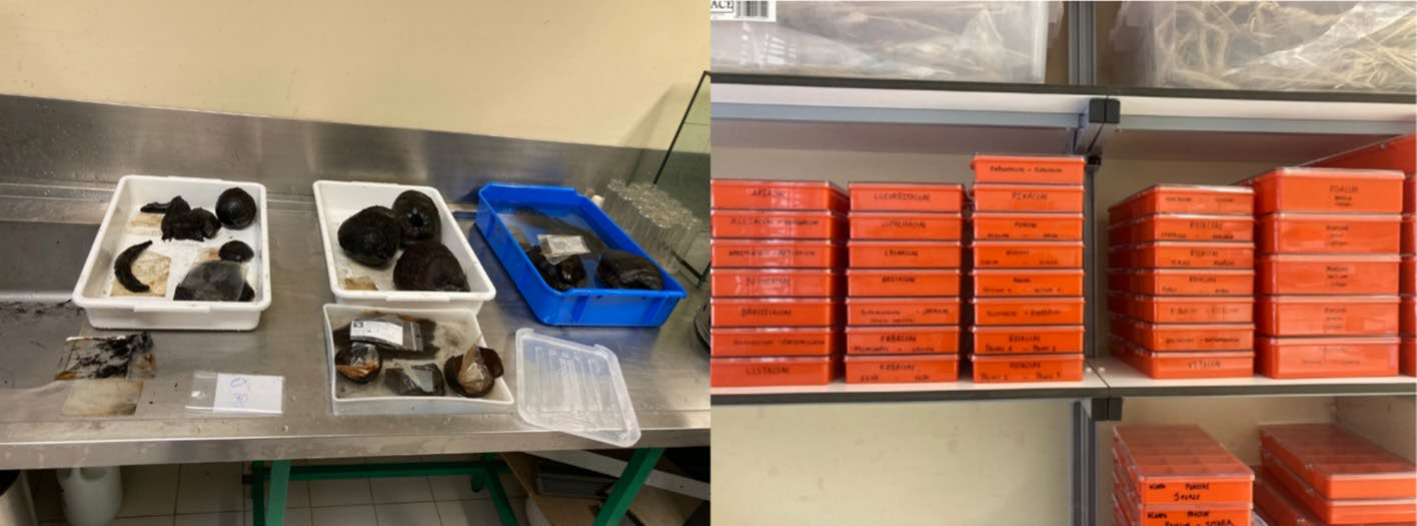
Washing of archaeological plant materials (left) and reference collection (right)

For wet manual collections, laboratory processing followed straightforward procedures. Each field container was opened, and its contents were washed and sorted by taxon (Fig. 7). Species identification was carried out through comparison with botanical atlases (Sabato and Peña-Chocarro 2021; Neef et al. 2012), online databases (e.g., Flora-On; UTAD; Digital Plant Atlas), published articles, and reference collections (Fig. 7). All botanical nomenclature followed World Flora Online (https://www.worldfloraonline.org/).

Large remains were identified by direct observation. Smaller remains were examined under a binocular magnifier.

For each specimen, an inventory record was completed, including all field label information, sample number, individual ID, taxon, condition (whole or fragmented), and identified plant part. All large specimens were photographed to scale. For smaller taxa, a representative specimen per taxon was photographed using a binocular magnifier.

Post-analysis, materials were stored in sealed water-filled bags, placed in airtight boxes to conserve space, and prevent desiccation. Smaller remains were kept in microtubes, test tubes, or analytical containers with water. Only complete, unfragmented seeds or fruits were counted as units, halves were considered when at least one extremity of the fruit/seed was complete, everything else was considered a fragment. All collected materials were analyzed.

## Results and Discussion

### Plant Remains in their Archaeological Context

A total of 205 plant macroremains belonging to eleven taxa were identified on the three ships (Tables 1, 2, and 3). Boa Vista 1 yielded three remains from two taxa, Boa Vista 2 yielded 102 remains from six taxa, and Boa Vista 5 yielded 100 remains from seven taxa. Most remains derive from stratigraphic units associated with the direct hull fill or ballast deposits (see Tables 1, 2, and 3).

**Table 3:** – Identification of macroremains from the Boa Vista 5 vessel. SU stands for stratigraphic unit

Boa Vista 5																
SU	Sector	*Cocos nucifera*		*Theobroma cacao*		*Pinus pinaster* (pine cone)		*Pinus sp. (pine cone)*		*Cucumis melo*		*Juglans regia*		*Posidonia oceanica* (egagropilo)		Total number of macroremains
		Unit	Fragment	Unit	Fragment	Unit	Fragment	Unit	Fragment	Unit	Fragment	Unit	Fragment	Unit	Fragment	
1497	BV5		14													14
1488 D6	BV5 005		2													2
1488	BV5 007		4													4
1488 C5	BV5 008		2													2
1488 D6	BV5 010		9													9
1488 E6	BV5 011		1													1
1488 C6	BV5 017		2													2
1488 F8	BV5 031		4													4
1488 D11	BV5 034		2													2
1488 F6	BV5 038		1													1
	BV5 049		1													1
1488	BV5 050		1													1
1488 B6	BV5 052		1													1
1488	BV5 087		1													1
1488 G6	BV5 120		3													3
1494	BV5 146		10													10
1494	BV5 179		1													1
	BV5 191 e 192		1											1		2
	BV5 193		2													2
1487	BV5 197		3													3
1487	BV5 207	1	1													2
	BV5 25b		1													1
	BV5 261												1			1
	BV5 312		1													1
1487	C.3 BV5 006		1													1
1488	D6 BV5 130										1					1
1488	G2 BV5 085		1													1
1488	G2 BV5 121		3													3
1488	G3 BV5 122		1													1
1488	T4 BV5 082		1						1							2
	No info	1	13	4		1			1							20
	Total	**2**	**88**	**4**		**1**			**2**		**1**		**1**	**1**		100

**Fig. 8:**
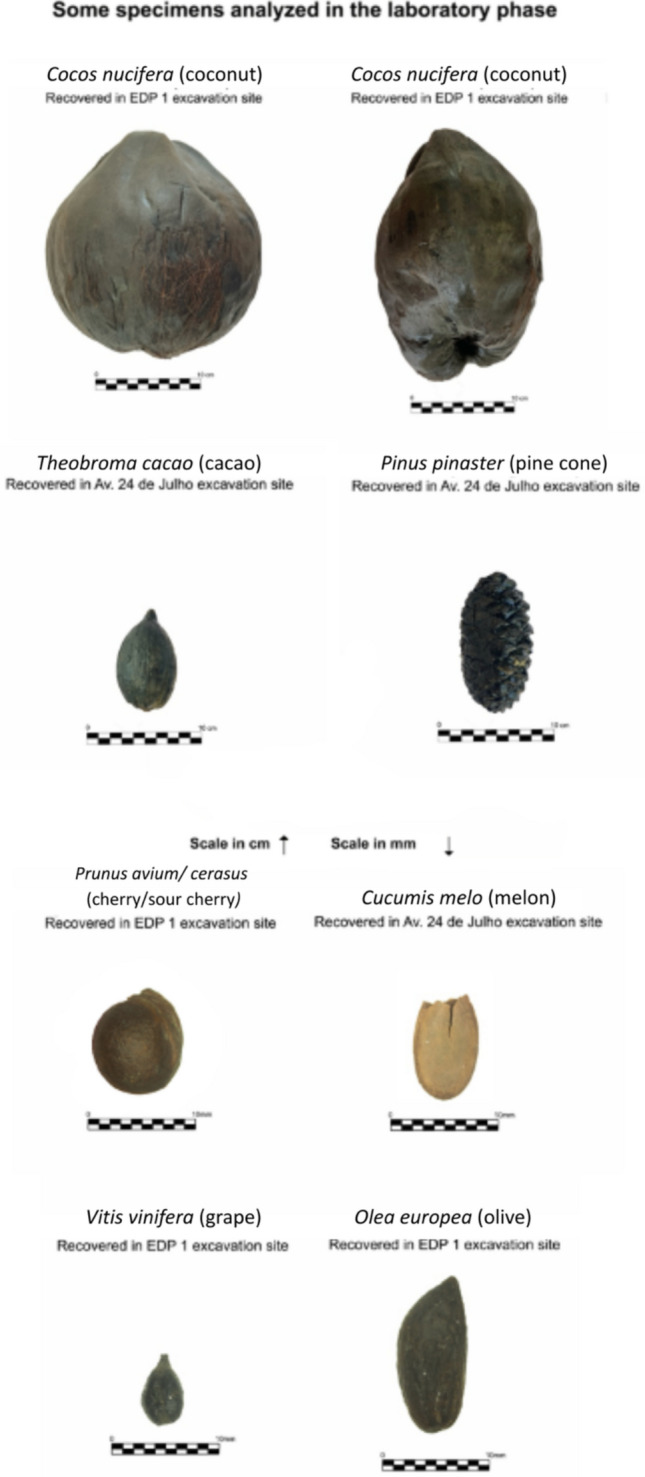
Examples of botanical material identified during sample processing, shown with individual scales to allow clear visualization of each specimen, considering their distinct sizes

Analysis of the collections from the three ships reveals that larger plant remains, particularly *Cocos nucifera* L. (coconuts), dominate the assemblage. The results are presented in Tables 1, 2, and 3. This is noteworthy for two main reasons. First, the fact that these ships were abandoned suggests that any high-value cargo had already been removed. Therefore, the presence of these coconuts and the other items left on board could indicate that they were of less economic importance. Second, the prevalence of large remains highlights a significant methodological bias resulting from the absence of systematic sediment sampling, which limits the recovery of smaller botanical materials and skews the diversity profile (see Fig. 8 for comparative sizes between species).

Clear differences were noted between the vessels. Boa Vista 1 yielded only two coconut fragments and a single *Olea europea* L. (olive pit), whereas Boa vista 2 and Boa Vista 5 yielded over one hundred individual specimens. Boa Vista 5 also showed slightly higher taxonomic diversity, although the limited quantity of most remains (excluding coconuts) complicates interpretation. Despite these constraints, the results demonstrate the coexistence of exotic and local products on board or in use on the Lisbon waterfront, such as pinecones (*Pinus pinea* L. and *Pinus pinaster* Aiton), melons *(Cucumis melo* L.*)*, cherries/sour-cherries (*Prunus avium* L./ *Prunus cerasus* L.), olives (*Olea europea* L.), grapes (*Vitis vinifera* L.), walnuts (*Juglans regia* L.), and coconuts (*Cocos nucifera* L.).

The abandonment context complicates efforts to distinguish between these two uses, as commercially valuable items were likely removed prior to abandonment. The limited number of individuals for most species and the above-mentioned recovery strategies prevent definitive conclusions regarding their representativeness in the archaeological record. The dominance of coconuts and other large remains is also a product of the hand-picking recovery strategy. Smaller remains, such as grape pips, cereal grains, or small fragments of dried fruits, are far less visible during excavation and therefore underrepresented. As a result, taxonomic diversity in all three vessels must be considered a minimum estimate rather than a full picture of plant use or cargo composition.

Another significant issue is the degradation of labels, which resulted in the loss of contextual data. For instance, four whole cocoa pods (*Theobroma cacao* L.) were recovered in the Boa Vista 5 context, but the lack of associated documentation prevents confirmation of their direct association to a specific part of the ship. However, the available data and testimonies from excavation team members, some of whom contributed to this article, support the identification of cocoa as part of the ship assemblage.

**Fig. 9:**
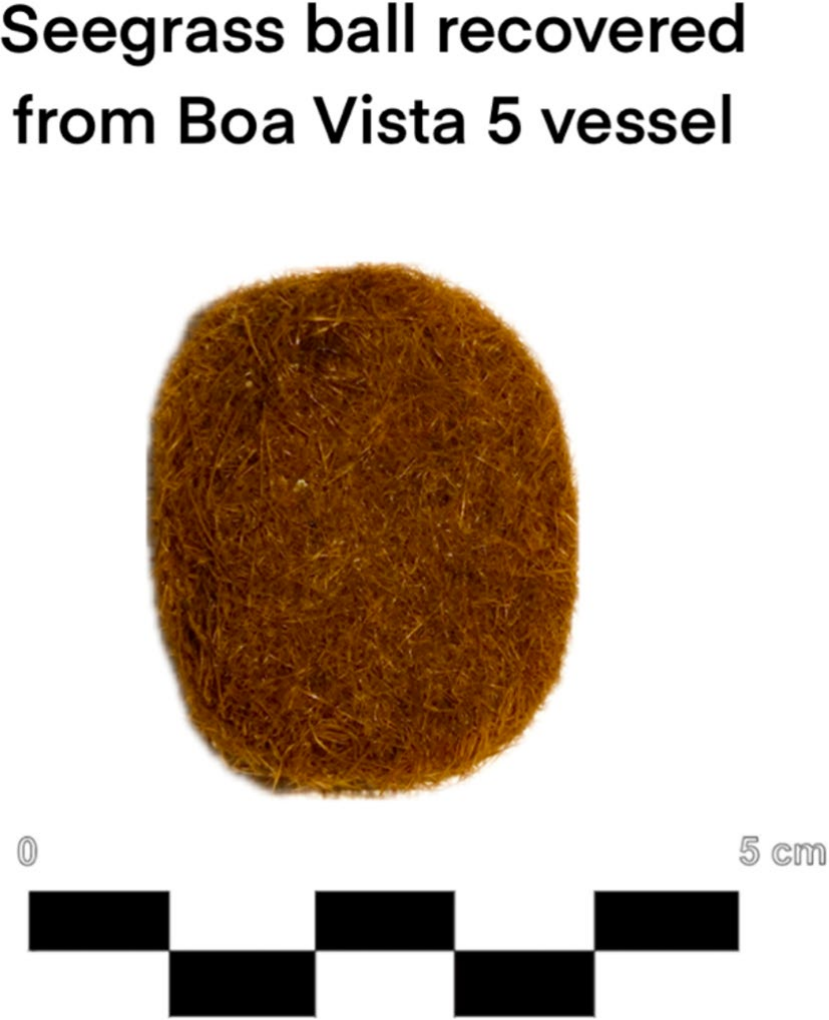
Specimen of *Posidonia oceanica* egragopilo identified in Boa Vista 5 vessel

A particularly intriguing find was a specimen of *Posidonia oceanica* L. egagropilo (seagrass balls) (Fig. 9), commonly formed from marine debris and known to accumulate along coastlines (Lefebvre et al. 2021).

### Historical and Economic Relevance of the Fruits

**Fig. 10:**
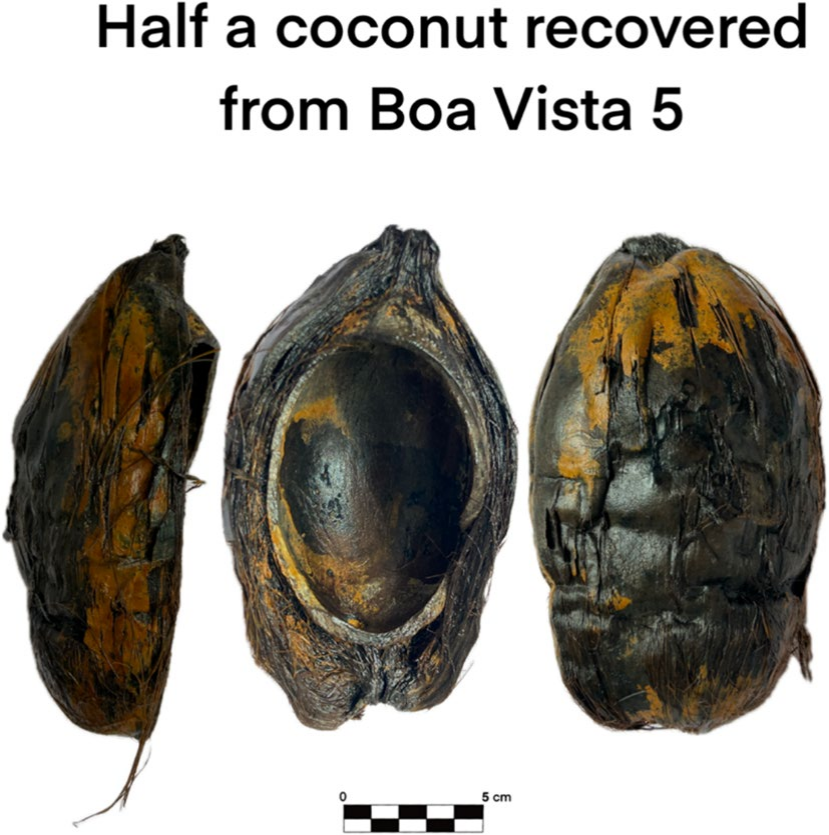
Detailed photograph of a coconut recovered from Boa Vista 5

**Fig. 11:**
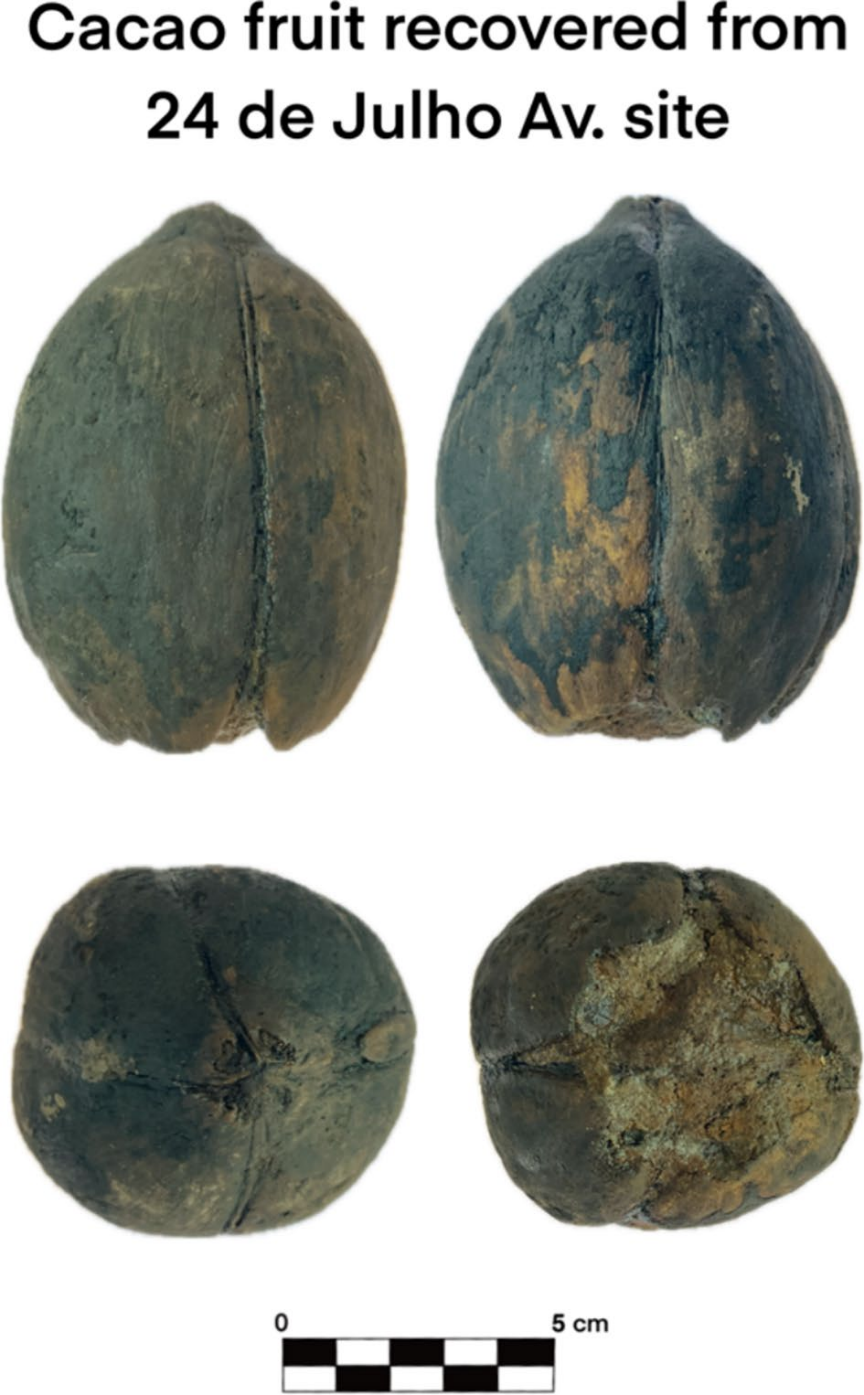
Detailed photographs of different angles of a cacao fruit recovered from *Avenida 24 de Julho* site

Given the prominence of coconuts (Fig. 10) and cacao (Fig. 11) among the finds, it is pertinent to briefly explore their historical and economic significance.

**Fig. 12:**
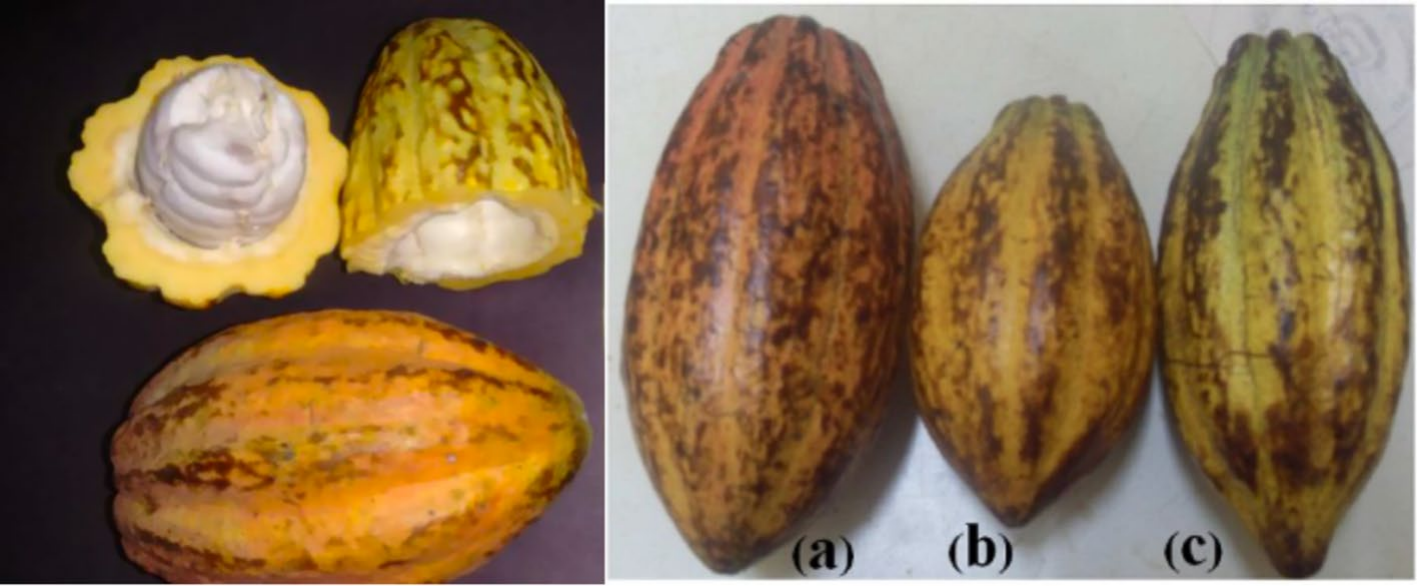
**a** Criollo; **b** Forastero; **c** Trinitario. Fresh cacao fruit/pod (De Souza et al. 2018) and different varieties of cacao (Josué et al. 2016)

Cacao (Fig. 12), native to Central and South America, was highly valued by Indigenous cultures such as the Aztecs, who used the fruits as currency and made a paste, that when solidified was consumed in rituals, as well as a beverage mixed with different ingredients. When introduced to Europe, cacao initially garnered attention for its cultural significance to the indigenous and as proof of the finding of new land. Roasting and grinding beans, like the indigenous already did, became a common practice to produce a bitter beverage, and later, with the altered recipe to include sugar, quickly became a luxury item across Europe (Ferrão 2005). In Brazil, however, Indigenous communities also made fermented beverages and jams from the pulp around the beans, a tradition that continues today with the production of cacao wine (Ferrão 2005). While cocoa remained a luxury import from the Americas, Portuguese efforts expanded its cultivation to São Tomé and Príncipe into the nineteenth century (Ferrão 2005). The roasting and grinding process to obtain the cacao powder was already described in recipe books in the seventeenth and eighteenth centuries, showing how these procedures were carried out in a domestic setting and not mass-produced like sugar (Freire 2020).

**Fig. 13:**
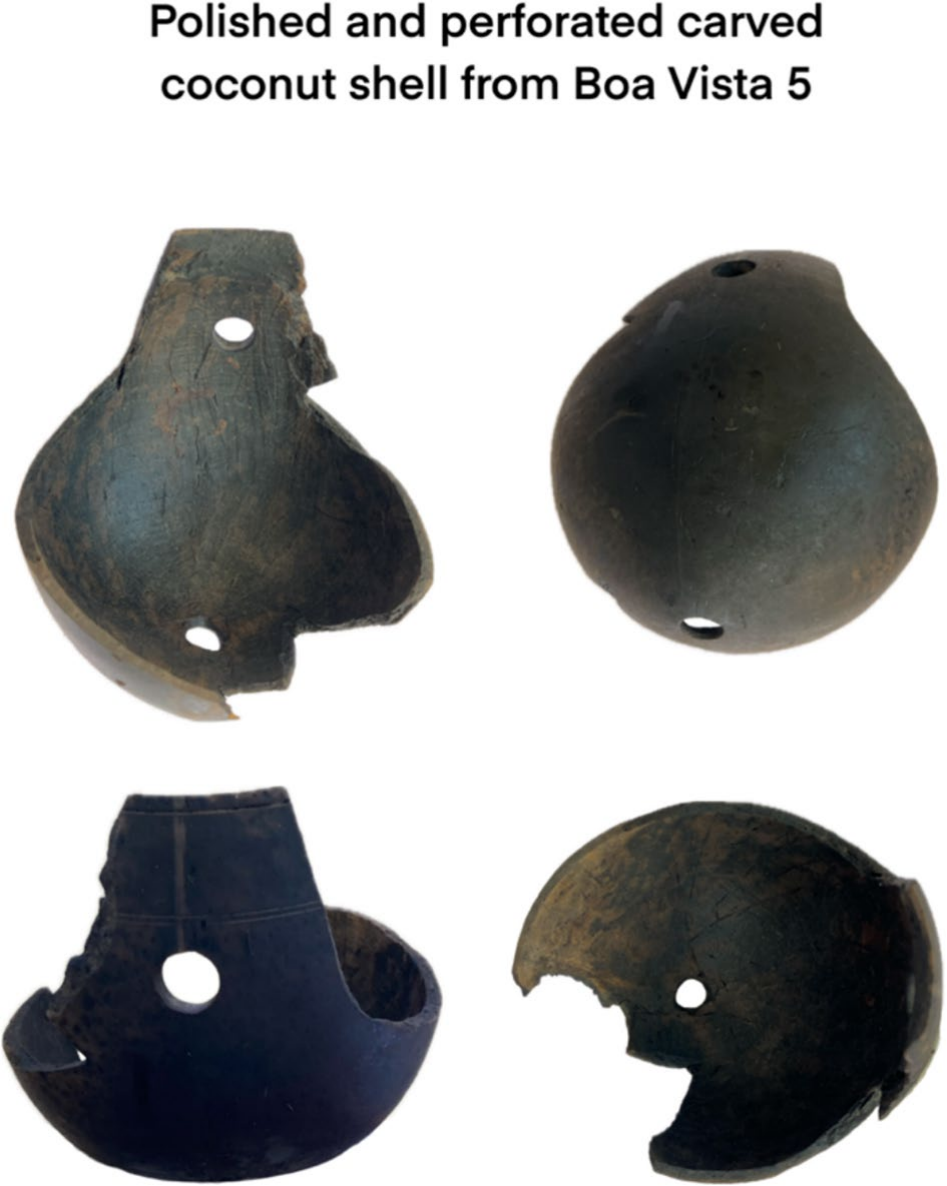
Example of the use of coconut shells to produce objects

The coconut, likely of Southeast Asian origin, was familiar to Europeans by the seventeenth century and widespread in tropical regions across the Americas and Africa. Portuguese explorers first encountered it during Vasco da Gama’s voyage to India. Valued as a multipurpose resource on long sea voyages, coconuts provided drinking water, food, fiber (used for ropes), and durable shells for utensils (Fig. 13). The first mention of coconut in a culinary recipe in Portugal is only known in the early eighteenth century (Freire 2020). Although it had been traded earlier, interest in coconuts grew significantly in the nineteenth century due to their use in lubricants and soap production (Ahuja et al. 2014). The presence of whole coconuts aboard abandoned vessels further suggests their intended use on board rather than as trade goods.

Other fruits identified, melon, cherry, grape, olive, pinecones, and walnut, have long histories in Europe, dating back to Roman times or earlier (Peña-Chocarro et al. 2019). Many of these could be preserved as dried fruits, jams, or through natural durability, making them ideal for long voyages and trade. Historical sources confirm their sale and consumption in Lisbon in preserved forms (Gomes 2017). Pinecones, on the other hand, could have been used for different purposes, for instance, as kindling, although those of *P. pinea* also provided a valuable food source.

The coexistence of these diverse products on board transatlantic vessels, during their final recorded voyages, provides tangible evidence of early plant globalization. These ships were not only transporting goods from Portuguese colonies but also facilitating the exchange of plant products across continents. In the specific case of the Boa Vista 2 and Boa Vista 5 ships, their presence shows that these ships were involved in transatlantic navigation, with a long area of operation that included connections to ports on the European, American, and Asian continents. There are not many studies that include an archaeobotanical approach when it comes to shipwrecks. A few examples would be *Vrouw Maria*, a Dutch merchant vessel from the eighteenth century, where botanical studies conducted confirmed the presence of coffee beans (*Coffea arabica* L.), indigo (*Indigofera tinctoria* L.), and madder (*Rubia tinctorum* L*.*), among others plant remains (Lempiäinen-Avci et al. 2022). *Gribshuden*, a Danish warship from the fifteenth century with a rich variety of plant remains, exotics, including saffron (*Crocus sativus* L.), cloves (*Syzygium aromaticum* L.), ginger (*Zingiber officinale* Roscoe), peppercorns (*Piper nigrum* L.), mustard (*Brassica nigra* L.), and dill (*Anethum graveolens* L.), and others, better known in the region, such as wheat (*Triticum* cf. *aestivum* L.), hazelnuts (*Corylus avellana* L.), almonds (*Prunus dulcis* (Mill.) Rchb.), and grapes (Larsson and Foley 2023). Finally, another example of a shipwreck where plant macro remains were identified is the Portuguese Indiaman *Nossa Senhora dos Mártires*, known as The Pepper Wreck, precisely because of the great number of peppercorns identified during its intervention (Castro 2003).

The presence onboard of *Posidonia oceanica* egagropilo remains ambiguous. Although it may have been collected incidentally, historical records suggest it was used as mattress filling in the early twentieth century, but its uses for the seventeenth century, if existent, remains uncertain (Royal Botanic Gardens, Kew 1906).

Despite limitations in sampling methodology, the physical presence of these botanical species aboard Early Modern ships supports broader narratives of global plant exchange and maritime provisioning. Sediment sampling would undoubtedly have yielded a richer dataset, yet even these limited remains contribute meaningfully to our understanding of Lisbon’s role in the early stages of global botanical circulation.

## Conclusion

The archaeological work carried out along Lisbon’s riverside has been essential in tracing the evolution of this space, from a natural beach to a highly modified urban-industrial zone. The landfills that pushed the coastline forward and the increasing port activity between the fifteenth and nineteenth centuries are more than just historical footnotes. They preserved organic remains that are rare in other urban contexts in Lisbon, namely plant macro remains, which are an important source for studying their introduction and use in a European context. They are physical layers that help us reconstruct the city’s role in the broader Atlantic world.

Boa Vista 2 and Boa Vista 5 ships, although not shipwrecks, offer an exceptional glimpse into the material life of port activity. Their abandonment, rather than catastrophic loss, connects them directly to the daily movement of goods, people, and practices, likely tied to the Junta do Comércio and Portugal’s Atlantic expansion. The poor conservation of Boa Vista 1, due to post-depositional processes, along with the absence of systematic sediment sampling, understandably limits the scope of our interpretations. Still, what was preserved, namely on Boa Vista 2 and 5, tells us a great deal.

The botanical remains recovered, particularly the large materials like coconuts and cacao, reflect both a methodological bias (handpicking naturally favors visible items) and the real circulation of both exotic and local products. Grapes, olives, melons, cherries, and nuts found alongside tropical goods like coconuts and cacao speak to a web of trade routes connecting Portugal to its colonies and beyond. Whether these goods were being consumed by the crew or destined for trade is difficult to determine, especially in abandonment contexts where the most valuable items were likely removed. But the diversity itself is revealing.

Finds like *Posidonia oceanica* egagropilo raise interesting questions about shipboard life and potential reuse of coastal materials, even if we cannot say with certainty what it was doing there. Just as telling are the limitations: without labels, without complete documentation, we risk losing critical context. The degraded information surrounding the cacao pods from Boa Vista 5 is a good example of this loss.

In short, while the sample is small and shaped by clear preservation and methodological biases, the materials still offer a valuable, if fragmentary, window into the maritime and commercial life of early modern Lisbon. These finds remind us of the importance of improving sampling techniques in wet and organic-rich contexts, where so much of the past can still be physically present.

Equally important is the potential of combining different sources of evidence, archaeological remains, written documentation and iconography, to construct richer and more nuanced understandings of the past. This interdisciplinary approach is particularly promising for exploring the everyday lives of diverse social groups in the early modern period, revealing not just what was traded or transported, but how people lived, worked, and interacted within these evolving maritime networks. Far from being static artifacts, these ships and their contents become dynamic traces of a world in transformation.

## Data Availability

No datasets were generated or analysed during the current study.
